# Geographic distribution of HCV genotypes in Libya and analysis of risk factors involved in their transmission

**DOI:** 10.1186/s13104-015-1310-x

**Published:** 2015-08-21

**Authors:** Mohamed A Daw, Abdallah El-Bouzedi, Aghnaya A Dau

**Affiliations:** Department of Medical Microbiology, Faculty of Medicine, Tripoli University, 82668 Tripoli, Libya; Libyan National Surveillance Studies of Viral Hepatitis & HIV, Tripoli, Libya; Department of Laboratory Medicine, Faculty of Biotechnology, Tripoli University, 82668 Tripoli, Libya; Department of Surgery, Faculty of Medicine, Tripoli Medical Center, Tripoli, Libya

**Keywords:** Libya, Hepatitis C virus, HCV subtypes, Genotypic variability, Africa

## Abstract

**Background:**

Hepatitis C virus (HCV) genotypes have been shown to be differently distributed between distinct geographical areas. Libya is a large country has the longest coast in the Mediterranean basin. Information regarding hepatitis C genotypes and subtypes circulating in Libya are not well known. The objectives of this study were to determine the frequency of various HCV genotypes cross Libya and the demographic and attributable risk factors associated with HCV transmission among Libyan population.

**Methods:**

A cross-sectional study was carried out on patients with recently confirmed HCV infection. A total of 3,227 serum samples enrolled at 19 collection center cross Libya. 1,756 belonged to Tripoli region, 452 to West region 355 to North region, 181 South regions and 483 East region. The samples were tested by type specific genotyping assay and correlated with demographic and potential risk factors within the studied populations.

**Results:**

A total of 20 discrete genotypes and subtypes were identified among the Libyan population ranging from 11.5 to 0.3 % cross the country. Genotype 1 was the most frequent among all regions (19.7–40.5 %), reaching the highest value in Tripoli region, followed by genotype 4 which was more prevalent in the South (49.3 %) and West (40.0 %) regions. Genotype 3, was higher in Tripoli (21.3 %) and East (15.9 %) regions while genotype 2, common in North (23.6 %) and South (22.5 %) regions. However, we found evidence that there is a changing relative prevalence of HCV genotypes in relation to age, gender and the mode of transmission which is reflected in the predominance of certain genotypes among Libyan population.

**Conclusions:**

Different HCV genotypes were isolated form Libyan population including newly emerged ones. The prevalence of the genotypes varied by geographic region and influenced by demographic and risk factors. Knowing the frequency and distribution of the genotypes would provide key information on understanding the spread of HCV in Libya and this could be greatly reflected on national plans and future strategies for infection prevention.

## Background

Hepatitis C virus (HCV) is a leading cause of chronic viral hepatitis, liver cirrhosis and hepatocellular carcinoma. Such implications have great, clinical, epidemiological and economic burdens worldwide particularly among developing countries [1, 2]. Relative risk factors as well as intervention and preventions programs are greatly influenced by regional variation of the HCV genotypes [3]. Genotyping plays an important role in the pathogenicity and hepatocarcinogenic potential of hepatitis C infection. Indeed pathological consequences and therapeutic responses has been greatly influenced by the HCV genotypes [4].

Different studies have shown that HCV genotype 1 has been associated with lower rates of response to peginterferon-based regimens compared with genotypes 2 and 3. Furthermore, infections with genotype 1a responds less than genotype 1b to treatment with peginterferon/ribavirin and to triple regimens that include telaprevir or boceprevir [5, 6]. Differences in pathogenicity and hepatocarcinogenic potential of the various genotypes of HCV were also reported. Raimondi et al. found that there was an increased risk of HCC in patients infected with genotype 1b, with or without the presence of cirrhosis [7]. Other studies also found both subtypes within genotype 1 to confer heightened risk of histopathological damage, concomitant progression to HCC, and to a lesser extent, persistence of infection [8, 9]. Many of these studies are from geographical regions where such genotypes predominate, hence then, further studies from different regions are needed.

The relative prevalence of HCV genotyping varies geographically and demographically worldwide. HCV genotypes 1a and 1b, are the most frequent genotypes in the North American and Europe [10, 11], and genotypes 5 and 6 seem to be confined to South Africa and Hong Kong [12, 13], respectively. HCV genotypes 7, 8, and 9 have been identified only in Vietnamese patients [14, 15] and genotypes 10 and 11 were identified in patients from Indonesia [16]. HCV genotypes and subtypes within the country also vary according to ethnicity and age. However, world integration and recent massive population displacement has lead to introduction of HCV genotype among a certain areas that not commonly known. HCV genotype distribution has been used for the identification of populations’ origin of HCV infection, examination of historical trends in characteristics of HCV infection and making inferences regarding routes of HCV transmission [17].

Hepatitis C virus infection is a major public health concern among African countries, they have the highest prevalence rates of HCV in the world, ranging from 1 to 26 % [2, 18]. Over 28 million people are chronically infected with HCV in this continent, and it is difficult to speculate about current and future trends [19]. HCV genotyping rarely studied in Africa, however, few studies were carried on a restricted populations in North Africa have shown diversity of HCV genotyping among these countries [20]. Therefore, population based studies are needed in order to plan for future prevention and intervention programs. Libya is the second largest country in Africa, with the longest coast facing the Southern European Union [21]. Bordering six different countries, where HCV has been considered to be endemic [22, 23]. In recent years the country has attracted worldwide attention because many Africans transit through it to enter Southern Europe illegally, with the possibility of transmitting infection in transit, or at the destination. Hence then a wide variation in the frequencies of HCV genotypes would be expected among Libyan territory.

In Libya HCV has been well studied, recently a comprehensive study including 1 % of the total population was carried. HCV prevalence ranged from 0.9 to 1.2 %. It was most prevalent among intravenous drug users (7.4 %) and less prevalent but still substantial in those undergoing blood transfusion (2.7 %), surgical operation (2.3 %) or hospital admission (1.9 %) [24]. However, few studies had lend themselves studying HCV genotype frequencies in small cohorts or restricted populations, but information concerning the distribution of viral genotypes and demographic and risk factors that influence HCV infection are limited [20]. The objectives of this study were aimed to determine the distribution of various HCV genotypes and subtypes present in different geo-graphical regions of Libya and the association with various risk factors involved in the transmission HCV infection among Libyan population.

## Methods

### Study population

Serum samples were collected from different patients with chronic HCV from all Libyan provinces based on the population proportion. Libya is a large country with a total geographic area; 1,775,500 km^2^ (square kilometers). The estimated Libyan population based on the consensus of 2006 was found to be 6,154,623 million. More than half of population were located in Tripoli region and less than a million in the Eastern region, the others are scattered over the vast areas of the country. A total of 3,227 different sera samples were collected from each of the five regions as following:

- Tripoli Region (TR) represented by five districts [number of isolates (n) = 1,756] including: Tajoura, Old City, Zwara, Alzawia, and Tarhona.
- West Region (WR) represented by three districts (n) = 452 including: Navosa Mountains, Garian and Ghadams.
- North Region (NR) represented by four districts (n) = 355 including: Sert, Musrata, Alkoms and Baniwaled.
- South Region (SR) represented by four districts (n) = 181 including: Aljofra, Ashati, Murzak and Sebha.
- East Region (ER) represented by three districts (n) = 483 including: Benghazi, Albtnan and Alkufra.

### Laboratory tests and HCV genotyping

All the serum samples were received along with specifically designed data sheets at Department of Medical Microbiology, Faculty of Medicine, Tripoli from 19 tertiary collection of the 5 regions involved. A written informed consent was taken from each patient and the data sheet contained demographic information including age, gender, year of diagnosis and risk factors for HCV such as history of blood transfusion, intravenous drug abuse (IVDA), history of surgical intervention, family history of HCV positive and history of promiscuity and dental procedure. Those patients who denied any risk factors were assigned to be as an; unknown group. Each serum specimen was re-tested positive for HCV antibody (anti-HCV) using 3rd generation commercial Enzyme Linked Immunosorbent Assays as previously described by Daw and El-Bouzedi [24]. HCV genotyping was carried out using type-specific HCV genotyping method [20]. All the different genotypes of HCV were tested and primers for genotypes 1a, 1b, 1c, 3a, 3c and 4 and 2a, 2c, 3b, 5a, and 6a primers were incorporated. Hence then HCV genotype for each sample was determined by identifying the HCV genotype-specific PCR band [20].

### Statistical analysis

Data were coded and entered into a data base, which was then cleaned and verified. Data were analysed by using the Chi square test with Yates’ correction or Student’s *t*-test for univariate analysis. The results for all variables were given in the form of rates (%). A multivariate analysis was conducted using logistic regression in order to verify which variables statistically had an influence on HCV infection such as gender (male vs. female), IV drug abuser (yes or no), blood transfusion (yes o r no) surgical Intervention and blood transfusion (yes or no), dental care (yes or no); promiscuity (yes or no). The data were analyzed using SPSS version 11.5 to identify the distribution of different genotypes and it s association with gender, age, year of diagnosis and risk factors [20].

### Ethical approval

The study was approved by the Libyan National Ethical Committee (Approval No. LY NS; HCV-G-399773). It was conducted in accordance with the Helsinki Declaration [25] and under the supervision of the Faculty of Medicine, Tripoli, Libya. All participants signed an informed consent form witnessed by the local health office before collection of data and blood samples. The questionnaire used to collect demographic and epidemiological data was anonymous and linked to the blood sample tube only by a code.

## Results

A total of 3,227 anti-HCV positive sera were received from all the five regions of the country. Out of these 1,501 (46 %) samples were found positive by HCV qualitative PCR with viral load >500 IU/ml were tested by type-specific genotyping assay. Out of 1,501 patients, 976 (65 %) were males and 525 (35 %) were females with an average age of 37.8 ± 13.4 years. Total of 817 (54.4 %) belongs to Tripoli region, 161 (10.7 %) to North region, 210 (13.9 %) West region, 71 (4.7 %) South region, 242 (15.1 %) East region. The distribution of HCV genotypes was shown in Table 1; a total of 20 different HCV genotypes and subtypes were found among the Libyan population. The distribution of such types was found to be as follows: 371 (11.5 %) were genotype 4; 219 (6.7 %) genotype 1; 205 (6.35 %) genotype 1b; 199 (6.17 %) genotype 3a; 135 (4.18 %) genotype 1a; 118 (3.66 %) genotype 2; (82 %) genotype 2a/c; 68 (2.11) genotype 4c/d; 40 (1.24 %) genotype 3;19 (0.59 %) genotype 4h; 15 (0.46 %) genotype 2b; 10 (0.31 %) genotype 2a; 7 (0.22 %) genotype 4e; and 4 (0.12 %) genotype 4a. Few stains were isolated at a low count (0.3 %) these include genotype 4b, 4f, 4h, 4d, 4a/c and HCV genotype 5 though genotype 6 was not reported.

**Table 1:** Distribution of HCV genotypes and subtypes among Libyan populations

Type/subtype	Total no of isolates (%)	Distribution among (%)	
		Male	Female
1	219 (6.79)	160 (7.26)	59 (5.77)
1a	135 (4.18)	103 (4.67)	32 (3.13)
1b	205 (6.35)	110 (4.99)	95 (9.29)
1a/b	3 (0.09)	2 (0.09)	1 (0.10)
2	118 (3.66)	72 (3.27)	46 (4.50)
2a	10 (0.31)	4 (0.18)	6 (0.59)
2b	15 (0.46)	8 (0.36)	7 (0.68)
2a/c	83 (2.57)	49 (2.22)	34 (3.32)
3	40 (1.24)	33 (1.50)	7 (0.68)
3a	199 (6.17)	170 (7.71)	29 (2.83)
4	371 (11.50)	216 (9.80)	155 (15.15)
4b	4 (0.12)	1 (0.14)	0 (0.10)
4e	1 (0.03)	6 (0.05)	1 (0.00)
4F	7 (0.22)	1 (0.27)	0 (0.10)
4h	19 (0.59)	8 (0.36)	11 (1.08)
4a/c	1 (0.03)	0 (0.00)	1 (0.10)
4c/d	68 (2.11)	29 (1.32)	39 (3.81)
4d	1 (0.03)	1 (0.05)	0 (0.00)
5	1 (0.03)	1 (0.05)	0 (0.00)
Unknown	1,726 (53)	1,227 (55.67)	499 (48.78)
Total	3,227 (100.00)	2,204 (100.00)	1,023 (100.00)

**Fig. 1:**
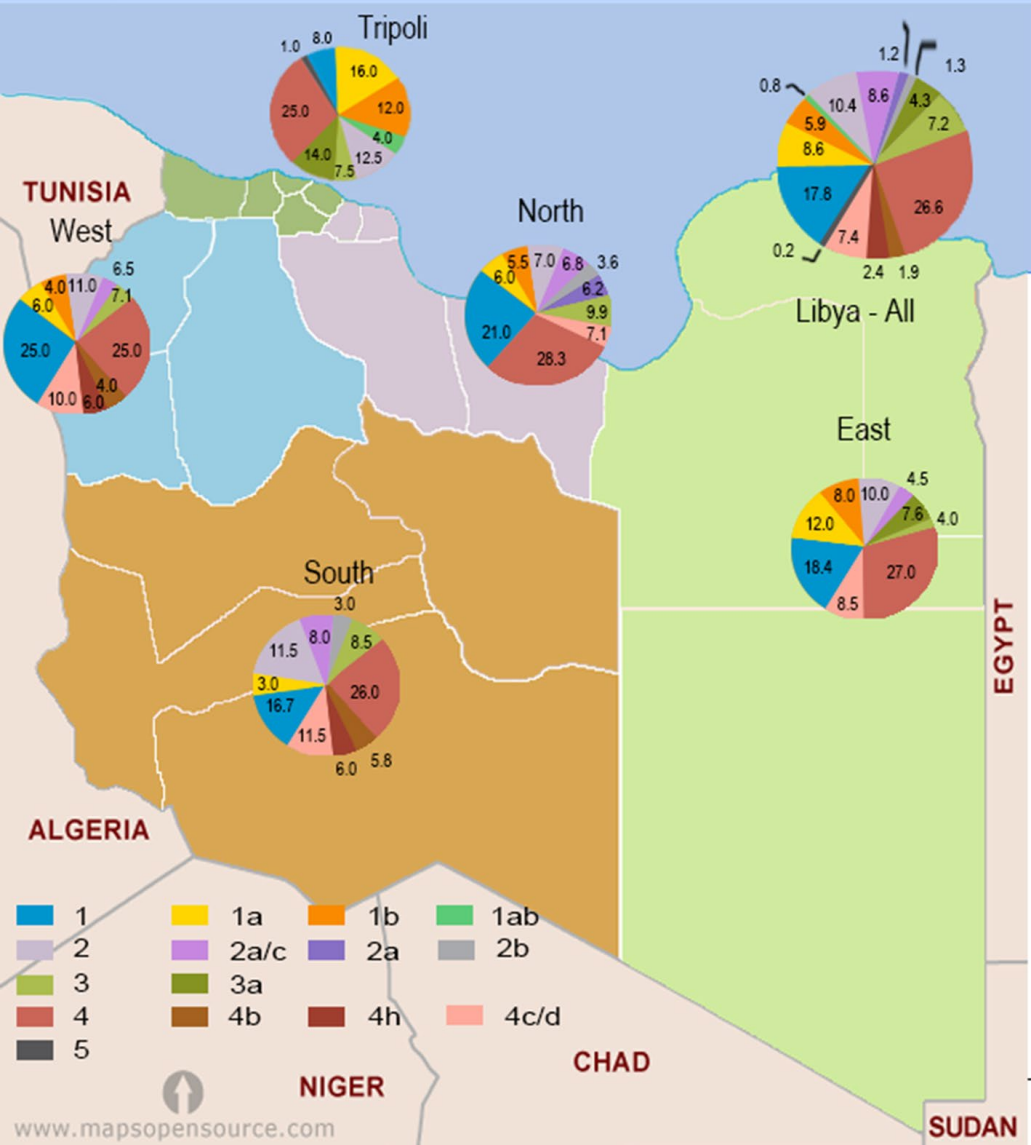
Geographical distribution of HCV genotypes according to the Libyan regions (*color*-coded). Data indicate percentage of patients infected by genotype in each region.

HCV genotype 1, 2, 3 and 4 were isolated from all over Libya, Fig. 1 shows the distribution of such genotypes according to the patients residential regions. Genotype 1 is the most common genotype isolated from Tripoli (40.5 %) and East regions (38.4 %), followed by the West (35.2 %) and North (31.1 %) regions and less reported in the South region (19.7 %); Genotype 4, South (49.3 %) and West region (40.0 %), followed by both East and North (35.5 %) and less among Tripoli region (25.9); Genotype 3, Tripoli (21.3 %) and East (15.9 %) and varied from 7.1 to 11.6 % among other regions; Genotype 2, North (23.6 %) and South (22.5 %) and from 12.2 to 14.5 % among other regions. HCV genotypes were found to be variables among the Libyan region involved (*P* < 0.26) though no variation within the provinces of each region.

**Fig. 2:**
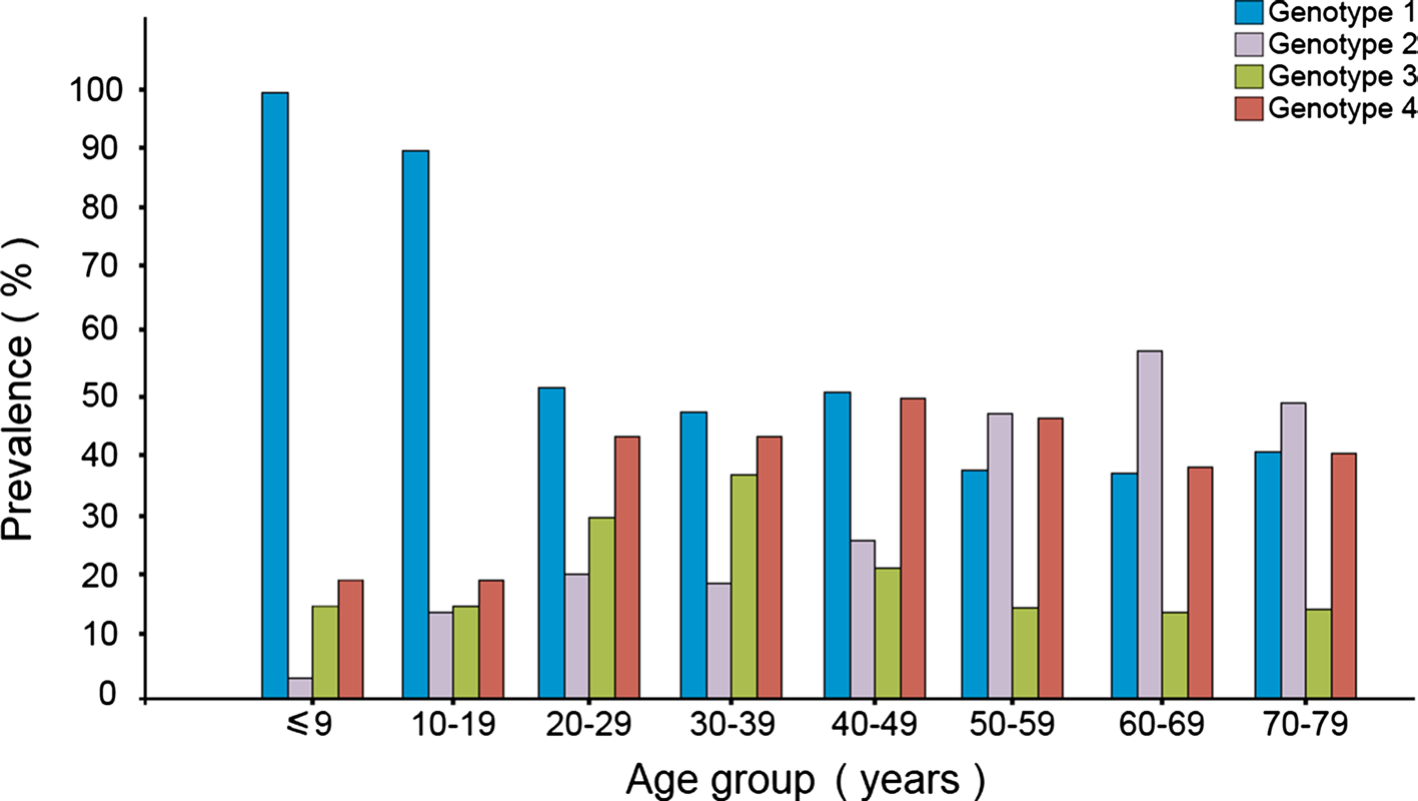
The distribution of HCV genotypes among different age groups of Libyan populations.

Table 2 illustrates the association between HCV genotypes and demographic variables (i.e. age distribution) among Libyan population. HCV genotype 1 was more frequent genotype among all age groups ranged from 25.3 to 88 %, followed by genotype 4 ranged from 8 to 37.6 % while genotype 2 and 3 are less frequent ranged from 0 to 45.5 % and 4 to 25.5 % respectively. Furthermore, HCV genotype 1 was the highest among those below (<20 years); 88 % within (0–9 years) and 78 % of (10–19 years). HCV genotype 2 was three times (OR = 0.2; 95 %CI 0.1–0.5, *P* < 0.001) higher among those aged over 50 comparable to a younger age population. Genotype 3 was twice higher among those aged 20–40 year of age than the other age groups. Figure 2 shows the distribution of HCV genotypes among different age groups.

**Table 2:** The distribution of HCV-genotypes among different age group

Age group	Genotype 1	Genotype 2	Genotype 3	Genotype 4	Total
0–9	22 (88.0)	0 (0.0)	1 (4.0)	2 (8.0)	25
10–19	46 (78.0)	2 (3.4)	2 (3.4)	9 (15.3)	59
20–29	113 (39.6)	29 (10.2)	53 (18.6)	90 (31.6)	285
30–39	199 (35.3)	44 (7.8)	144 (25.5)	177 (31.4)	564
40–49	109 (38.0)	41 (14.3)	29 (10.1)	108 (37.6)	287
50–59	40 (25.8)	55 (35.5)	6 (3.9)	54 (34.8)	155
60–69	25 (25.3)	45 (45.5)	3 (3.0)	26 (26.3)	99
70–79	8 (29.6)	10 (37.0)	1 (3.7)	8 (29.6)	27
Total	562 (37.4)	226 (15.1)	239 (15.9)	474 (31.6)	1,501

**Fig. 3:**
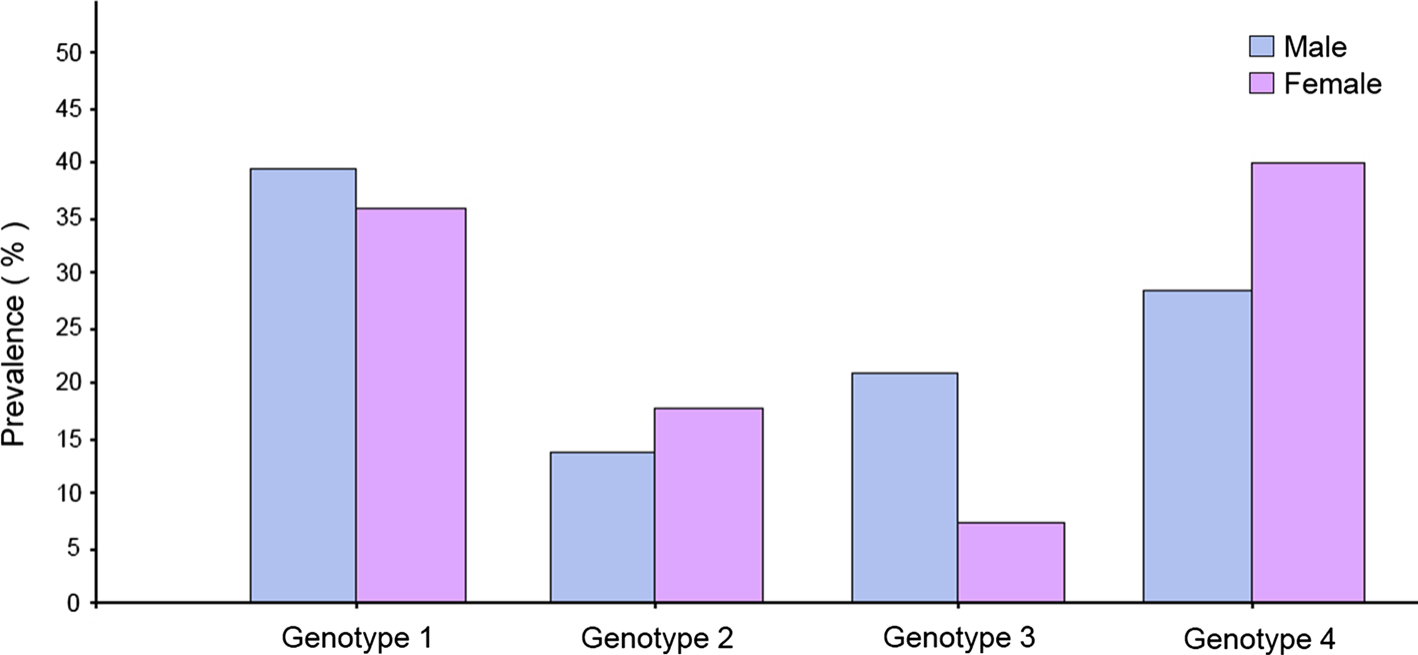
The influence of gender on the distribution of HCV genotypes among Libyans.

**Fig. 4:**
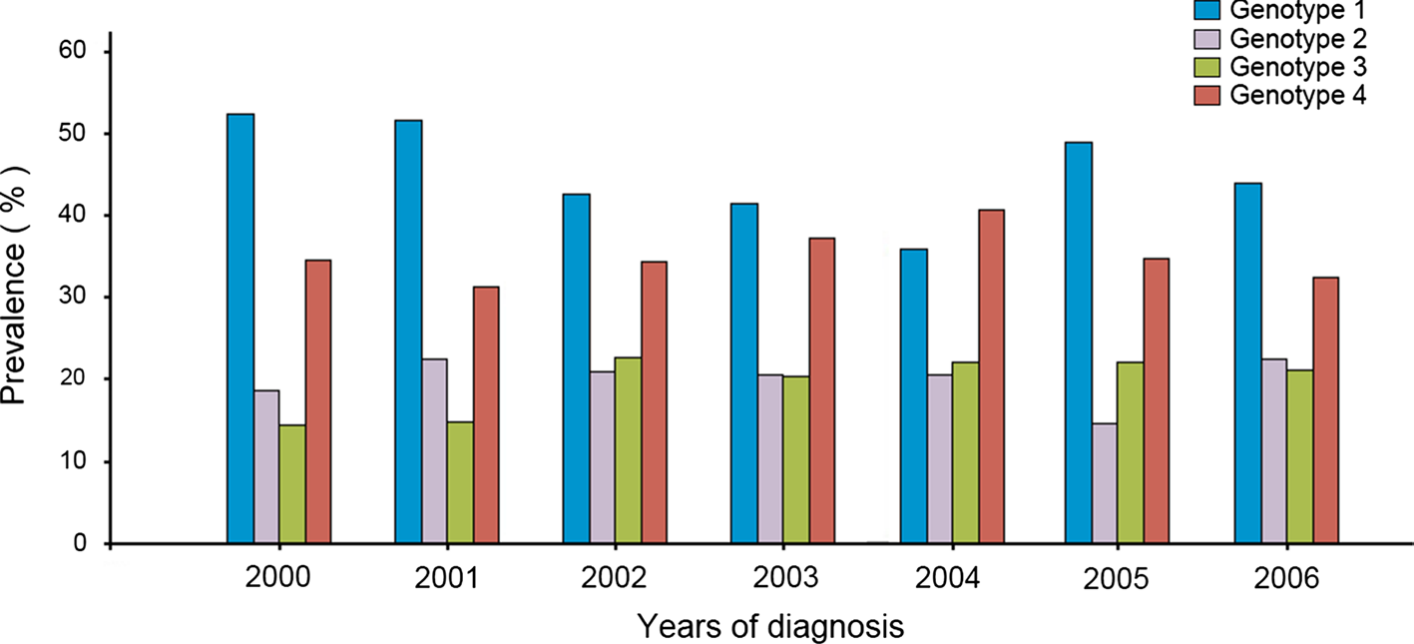
Distribution of hepatitis C genotype during the period of 2000–2006.

Figure 3 shows the influence of gender on the distribution of HCV genotypes among Libyans. Hepatitis C virus genotypes, 1, 4 and 3 are the common among both sexes. HCV-genotype 1 is highest among males (38.4 %), while genotype 4 is the highest among females (39.7 %). HCV subtypes were also influenced by gender variations. Subtype 1a, 1b, 1a/b, subtype 2a/c, 3a, 4a, 4b, 4f were found to be more common among males. Though subtype 2a, subtype 4h, 4a/c and subtype 4c/d were common among females. Hepatitis C genotypes vary from 1 year to another as shown in Fig. 4. Genotypes 1 and 4 we the commonly isolated one during the study period while genotypes 2 and 3 are slightly variable (*P* > 0.0025).

Table 3 shows the correlation between HCV-genotypes and the risk factors involved in the populations studied. HCV genotype 2 and genotype 3 were more frequent among intravenous drug abuse (IVDUs) comparable to non-users (non-IVUDs)-(IVUDs vs non-IVUDs) as it was found to be (49.2 vs 36.3 %) and (32.6 vs 14.3 %) respectively. Genotype 4 was more frequent among caesarean section comparable to those not operated (50 vs 14.3 %; *P* < 0.0001). Non of other risk factor were found to be significantly associated with any of HCV genotypes (*P* < 0.26). Multiple risk factors were identified among 9.5 % (6.1–2.3 % among the different age groups) of the subjects, where 7.4 % of them had two risk factors and 2.1 % had three risk factors. There are no risk factors found in 40.9 % of the patients under the study ranging from 37.1 to 51 % in the different age groups.

**Table 3:** Prevalence of HCV Genotype according to risk factors among Libyan population, * *P* = 0.26

Risks exposure	Genotype 1	Genotype 2	Genotype 3	Genotype 4	Total	*P* value*
Sexual						
No	551 (37.3)	225 (15.2)	234 (15.8)	467 (31.6)	1,477	0.424
Yes	11 (45.8)	1 (4.2)	5 (20.8)	7 (29.2)	24	
Cesarean section						
No	549 (37.7)	222 (15.2)	235 (16.1)	451 (31.0)	1,457	0.26
Yes	13 (29.5)	4 (9.1)	4 (9.1)	23 (52.3)	44	
Intravenous drug use						
No	497 (36.3)	226 (16.5)	196 (14.3)	450 (32.9)	1,369	0.000
Yes	65 (49.2)	0 (0)	43 (32.6)	24 (18.2)	132	
Blood transfusion						
No	420 (37.1)	167 (14.8)	196 (17.3)	348 (30.8)	1,131	0.071
Yes	142 (38.4)	59 (15.9)	43 (11.6)	126 (34.1)	370	
Surgery						
No	457 (38.1)	171 (14.3)	198 (16.5)	372 (31.1)	1,198	0.165
Yes	105 (34.7)	55 (18.2)	41 (13.5)	102 (33.7)	303	
Dental procedure						
No	490 (37.5)	193 (14.8)	204 (15.6)	418 (32.0)	1,305	0.641
Yes	72 (36.7)	33 (16.8)	35 (17.9)	56 (28.6)	196	
Intrafamilial						
No	549 (37.4)	222 (15.1)	232 (15.8)	463 (31.6)	1,466	0.876
Yes	13 (37.1)	4 (11.4)	7 (20.0)	11 (31.4)	35	
Haemodialysis						
No	551 (37.5)	225 (15.3)	234 (15.9)	460 (31.3)	1,470	0.185
Yes	11 (35.5)	1 (3.2)	5 (16.1)	14 (45.2)	31	

## Discussion

Hepatitis C virus genotyping has been found to play an important role in understanding the epidemiology, guiding the clinical therapy, vaccine development and assessing the risk–benefit of HCV infection [26–28]. In recent decades there has been a global epidemic of some of these genotypes and subtypes assembled with certain risk factors and modern medical practice [3, 4]. Regional differences appear to exist in the distribution of HCV genotypes. Such variability are rarely studied in Africa where habitual and ethnic variation exist and HCV is considered to be endemic [29]. Hence then studying distribution of various genotypes in this continent is essential for its prognostic implications in chronic hepatitis C infection.

In this cross-sectional epidemiologic study, with stratified sampling we investigated the distribution of various HCV genotypes in Libya, and the potential links with demographic and transmission risk factors. Our results indicate that HCV genotype 1 (37.4 %) is the most prevalent genotype cross Libya followed by genotypes 4 (31.6 %) and to less extent genotypes 3 and 2 and infrequent cases of genotype 5. This however, is in agreement in previous studies carried by our group though with a lower population size [20]. A substantial regional differences appear to exist in the distribution of HCV genotypes cross Libya. Genotype 1 and 3 were higher in Tripoli, and East regions though less among the others particularly south regions. Genotype 4 was higher in South and West regions while genotype 2 more prevalent in North and South regions. Such data correspondence well with other studies from different countries that have shown the genotypic regional diversity of HCV [30]. Genotypes 1 and 3 of Tripoli region were found to be similar to those in south Italy and Greece [31, 32]. Though genotype 4 of the South and East regions were also common in Egypt and other neighboring African countries [33]. Genotypes 2 and 3 were found to be more frequent in the South and North regions may suggest presence of foreigners living illegally in Libya, due to uncontrolled borders. Libya, however, is considered to be biggest seat for illegal transition immigration to EU particularly during the recent uprising and uncontrolled armed conflicts [19, 34]. Thus knowledge on the distribution of various genotypes in such country is essential for its prognostic implications in chronic hepatitis C infection. Hence then further study are needed to document the changes of HCV genotypes variability over periods of time.

The distribution of HCV genotypes in this study was influenced by gender. Genotypes 1 (M:F; 38.4:35.7 %), 3 (20.8:6.9) and 4 (17.6:14) were higher among males. Although few distinct subtypes within genotype 4 were more predominant among females particularly 4h (0.36:1.08) and 4c/d (1.32:3.81) while genotype 2 was more frequent among females (13.6:17.7). Our results are in concordance with a recently published data from other countries including Italy and Germany [35, 36]. However, most of those participated in the study were males (65:35). This may reflect the conservative nature of Libyan society and highlights the need to improve sampling techniques particularly among women sectors in the upcoming surveys.

Considering the age group distribution, the present study indicates that HCV genotype distribution varies with age. HCV genotype 1 is highly predominant among younger patients (<20 years). It reached 88 and 78 % among those aged (0–9 years) and (10–19) respectively. Genotype 2 was tripled among those aged above 50, although genotype 3 was significantly higher among those aged 20–40 years. Genotype 4 was evenly distributed in all age groups particularly over 20 years old. Different studies have analyzed the correlation of HCV genotypes in relation to the patients age. Similar results were also reported in other North African countries. In Algeria, genotype 1 is associated with the age group younger than 60 years though other genotypes types are higher among older age groups [37]. The frequency of genotypes and their variation according to age groups were clearly evident among the Libyan populations which was not reported before and thus appears to confirm the ‘stratification’ of genotypes associated with risk factors reported in the literature [2, 19, 20].

Different studies have suggested HCV genotypes may be associated with different transmission routes and high risk behaviors. Transmission by IVDUs is most common in Libya, and has become more frequent and associated with genotype 1 (49.2 %) and genotype 3 (32.6 %) infections. Studies from neighboring countries also found an association between genotype 1 and IVDUs but not genotype 3. However, it has also been reported that HCV genotype 3 is particularly prevalent in intravenous drug abusers in Europe and the United States due to wide spread use of heroin [38, 39]. The identification of genotype 3 among Libyan population reflected its worldwide occurrence among PWID. Further studies are needed to highlight the spread of this genotype particularly among the young people. Genotype 4 was significantly associated with Caesarian section (52.3 %) and intra-family transmission (31.4 %). Both genotype 1 and 4 were the predominant genotypes among medical practice including blood transfusion (1:4; 38.4:34.1), surgical practice procedures (34.7:33.1), dental practice (33:18.2) and haemodialysis (45.8:45.2). This is in agreement with other studies from different countries where majority of the cases with genotype 1 has a history of hospitalization for major/minor surgery, dental procedures, received blood and/or blood products and shaving by barbers [40–42].

In this study, over 15 HCV subtypes were identified including newly emerged ones such as 1a/b, 4b, 4e, 4f, 4h, 4a/c, 4d, 4c/d and HCV genotype 5. The distribution of these subtypes among the Libyan regions and their association with demographic and behavioral correlates were explored. Subtypes 1a, 1b and 3a are more predominant in Tripoli region (i.e. Old City and Bosleam) and East region (i.e. Benghazi) particularly among young males (<30 years) with history of IVDUs. This is in an agreement with other studies from America and Southern Europe countries who also found a higher percentage of individuals infected with HCV subtypes 1a, 1b, and 3a, are among IVDUs [43, 44]. HCV subtypes 4a, 4e, 4h and 4c/d more frequent in South and East regions of Libya and associated with hospital and modern medical practices. Genotype 4 is considered to be endemic in North Africa and Meddle-East and rarely reported in other regions and thus more likely to be associated with common public-health and family practices. However, further studies are needed to highlight the association of these subtypes with education levels and living standards conditions among such countries [3, 19].

Our study mirrors clearly the HCV genotypic diversity among Libyan populations. Such, genotyping profile variability may reflect differences in the timing of acquisition of HCV infection that may in turn influence the timing of the peak burden of HCV complications including cirrhosis and hepato-cellular carcinoma. As a consequence of these trends, regional differences in HCV genotype prevalence and epidemiology may warrant consideration of prevention and treatment strategies that are tailored to local needs [45].

The main limitations of this study that the correlation between HCV genotypes and the severity of the diseases among the studied population is lacking. Previous reports have shown that evidence of advanced liver disease and increased risk of hepatocellular carcinoma were more common in patients with HCV genotype 1 and 1b than other genotypes [46, 47]. Hence then future studies es are recommended to highlight such correlation particularly among naïve patients [48]. Additionally this study did not identify patients with possible HCV/HIV co-infection and HCV genotypes involved. Different studies have shown that the distribution of HCV genotypes in the HIV-infected population reflects the route of transmission and progression to both AIDS and death [49]. Genotype 1b associated with post-transfusion HIV infections and genotypes 1a and 3a are more common in intravenous drug users infected with HIV [50]. Furthermore, genotype 1 and 3 were associated with severe histopathological and steatohepatitis findings in HIV/HCV-co-infected patients and leads to significantly faster HIV progression [51].

## Conclusion

In conclusion regional difference in HCV genotypes were clearly evident among Libyan population with emerging of new genotypes, though genotypes 1 and 4 are more dominant. The frequency of these genotype are significantly associated with demographic and risk factors involved. Furthermore, the spread of intravenous drug use could have led to a new wave of HCV infection among young people. Therefore, a future evaluation of new ‘emerging’ subtypes over a period of time will be necessary, particularly nowadays as the country passing by difficult situations resulting in a massive population displacement and immigration engulfment [52].
